# Chronic Maxillary Sinusitis Associated with an Unusual Foreign Body: A Case Report

**DOI:** 10.1155/2012/903714

**Published:** 2011-10-13

**Authors:** Yunus Feyyat Şahin, Togay Muderris, Sami Bercin, Ergun Sevil, Muzaffer Kırıs

**Affiliations:** ^1^Division of Otolaryngology, Batman Şifa Hospital, 72070 Batman, Turkey; ^2^Department of Otolaryngology, Head and Neck Surgery, Ataturk Education and Research Hospital, Bilkent, 06800 Ankara, Turkey

## Abstract

Foreign bodies in maxillary sinuses are unusual clinical conditions, and they can cause chronic sinusitis by mucosal irritation. Most cases of foreign bodies in maxillary sinus are related to iatrogenic dental manipulation and only a few cases with non-dental origin are reported. Oroantral fistulas secondary to dental procedures are the most common way of insertion. Treatment is surgical removal of the foreign body either endoscopically or with a combined approach, with Caldwell-Luc procedure if endoscopic approach is inadequate for visualisation. In this case, we present a 24-year-old male patient with unilateral chronic maxillary sinusitis due to a wooden toothpick in left maxillary sinus. The patient had a history of upper second premolar tooth extraction. CT scan revealed sinus opacification with presence of a foreign body in left maxillary sinus extending from the floor of the sinus to the orbital base. The foreign body, a wooden toothpick, was removed with Caldwell-Luc procedure since it was impossible to remove the toothpick endoscopically. There was no obvious oroantral fistula in the time of surgery, but the position of the toothpick made us to think that it was inserted through a previously healed fistula, willingly or accidentally.

## 1. Introduction

Rhinosinusitis is an inflammatory process involving the mucosa of the nose and one or more sinuses, and usually more than one sinus is affected [1]. Unilateral maxillary sinusitis can be caused by various diseases, such as those affecting the teeth, fungal infections, trauma, tumors, or foreign bodies [2, 3]. Maxillary sinusitis secondary to the presence of foreign bodies in the interior of the maxillary sinus is an unusual clinical entity. Most cases of maxillary sinus foreign bodies in literature are related to iatrogenic dental manipulation [4]. Foreign bodies of very different nature, such as fillings, tooth roots, fragments of broken parts, or different types of implants, are introduced into the maxillary sinus by different mechanisms, such as apical migration of fragments of fillings or accidental rough handling.

Far rarer of maxillary sinus foreign bodies are nondental origin. Foreign bodies may be introduced willingly by the patient or accidentally usually through an oroantral fistula [5]. Oroantral communications are rare complications of oral surgery, which recognize upper molars extraction as the most common etiologic factor (frequencies between 0.31% and 4.7% after the extraction of upper teeth) [6]. There is no agreement about the indication of techniques for the treatment of this kind of surgical complication. Spontaneous healing of 1 to 2 mm openings can occur, while untreated larger defects are connected with the pathogenesis of sinusitis [7].

Only a few cases of nondental paranasal sinus foreign bodies have been reported in the literature. This paper reports a case of chronic maxillary sinusitis secondary to the inoculation of a toothpick into the maxillary sinus probably through a previously healed oroantral communication which developed after second molar tooth extraction.

## 2. Case Report

A 24-year-old male patient was attended to our clinic with complaints of headache, nasal obstruction, halitosis, chronic purulent rhinorrhea from his left nostril, and postnasal drip for approximately three years. He received antimicrobial therapy for sinusitis three times, but his complaints persisted. On physical examination, he had a mild septal deviation to left, his left inferior turbinate was hypertrophic, and he had purulent and foul-smelling discharge from his left nostril. Nasal endoscopy revealed polyps and purulent secretion on left middle meatus. In his oral examination, there was no obvious dental problem (cavities, mobility, etc.), but his left upper second molar tooth was absent due to extraction for root abscess three years ago, yet there was no sign of oroantral fistula. He had no history of surgery for nasal or sinus pathologies, and he had no chronic medical condition.

**Figure 1:**
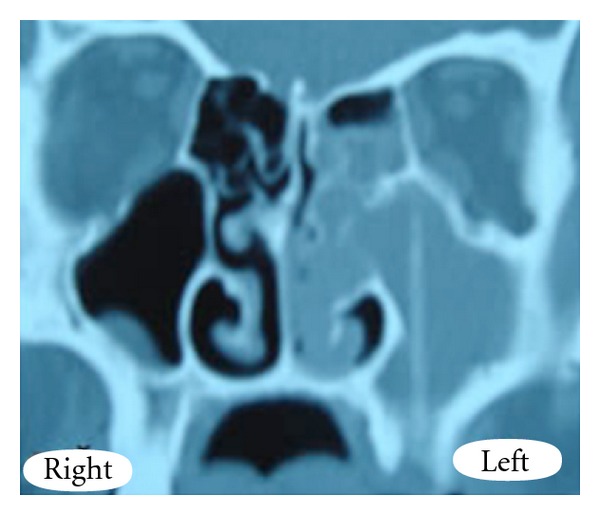
CT scan showing the foreign body in the left maxillary sinus.

A computed tomography scan of the paranasal sinuses was done prior to medical treatment because his sinusitis was unilateral and resistant to medical therapy. CT scan revealed sinus opacification with the presence of a foreign body in the left maxillary sinus extending from the floor of the sinus to the orbital base. (Figure 1) There was also a discontinuity in the bony segment of the sinus floor, but there was no sign of fistula since the connection between maxillary sinus and oral cavity was interrupted by soft tissues. Shape and position of the foreign body made us think that it had been inserted from a previously healed oroantral fistula, where the second upper molar tooth had been extracted. But we could not find out if it had been inserted willingly or accidentally, because the patient could not remember such an incident.

**Figure 2:**
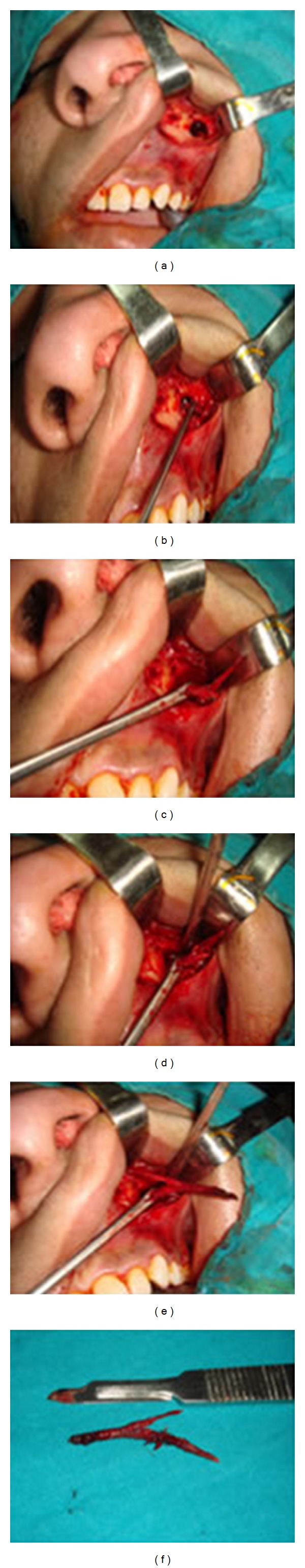
Removal of the wooden toothpick.

Endoscopic surgery was planned to take the foreign body out; left uncinectomy and middle meatal antrostomy were performed under general anesthesia. We saw the foreign body, a wooden toothpick, through the sinus ostium, and we realised that it was not possible to take out the toothpick endoscopically. So we turned the operation to an external approach using the Caldwell-Luc procedure for better visualization of the antrum. Toothpick was removed with the help of a forceps. (Figures 2(a)–2(f)) We checked oral cavity again for an oroantral fistula during surgery, but still there was no interruption in the oral mucosa which may suggest a fistula.

The patient was given antibiotics and topical decongestants for a week following surgery. The patient improved dramatically from the symptoms, and he was discharged the day after surgery without any complications. At the 1-year followup, the patients' physical examination and radiological investigations were normal.

## 3. Discussion

Foreign bodies in the maxillary sinus, whatever their origin, are rare entities, but they are an integral part of the differential diagnosis for rhinosinusitis, mainly when sinusitis occurs unilaterally. It is difficult to estimate their frequency because of the rarity of the entity, and because of the small numbers of series published. Although the exact mechanism of how foreign bodies cause sinusitis remains unknown, it has been suggested that foreign bodies produce chronic physical and chemical irritation of the mucosa, leading to a degree of ciliary insufficiency and secondary infection [8].

Maxillary sinus foreign bodies usually have a dental origin in relation to manipulation, or they may show up secondary to an oroantral fistula, as mentioned before. Oroantral communications occur most frequently following maxillary molar or premolar extraction. The surgeon should be extremely careful for inspecting oroantral communications especially after maxillary molar and premolar tooth extraction or endodontic surgery performed on maxillary teeth which may result in sinus perforation that may develop into oroantral communication more than into oroantral fistula [9, 10]. In this case, probably the oroantral communication which developed after maxillary premolar tooth extraction was very small, and the surgeon could not be able to recognize it during surgery. Then, after the insertion of the toothpick into the maxillary sinus willingly or accidentally, the communication is closed after a while with granulation tissue which occurred due to chronic irritation of the toothpick.

Because foreign bodies can cause irritation of the mucosa that can be concluded to sinusitis, the removal of all foreign bodies is generally recommended, even when they do not produce symptoms [11]. A foreign body can be removed with different techniques depending on the size and location of it. The most common technique is endoscopic sinonasal surgery allowing the removal of most foreign bodies via a wide endonasal meatotomy [12]. When extraction is not possible by the endonasal approach, it can be conducted through an external approach by oral antrostomy or a combined approach of endonasal meatotomy and oral antrostomy [13]. In this case, we used the combined technique because the size and position of the toothpick did not allow us to remove it from endonasal meatotomy.

This case is unusual and interesting for two reasons; first, the foreign body itself, a whole wooden toothpick, is the first in the literature to our knowledge; second, the position and direction of the toothpick made us think that it had been inserted through an oroantral fistula, which probably occurred secondary to a tooth extraction and healed after insertion of the toothpick without any surgical intervention. This case points out once more that it is very important to recognize and fix an oroantral communication occurred during a dental procedure immediately to prevent complications. Oroantral communications should be treated by establishing a physical barrier between oral cavity and maxillary sinus, and numerous surgical techniques have been introduced for repair, including rotating or advancing local tissues such as the buccal or palatal mucosa, buccal fat pad, submucosal tissue, or tongue tissue [9]. 

In conclusion, foreign bodies in the maxillary sinus are rare issues, and oroantral fistulas, which are usually secondary to dental procedures, are the most common way of insertion. Whatever the foreign body is, it must be removed to prevent chronic infections even if it is asymptomatic. Endoscopic sinonasal surgery shall be the choice of treatment, since it is minimally invasive, but combined approach with Caldwell-Luc procedure can also be used especially if endoscopic approach is inadequate.
